# 2,4-Dichloro-*N*-phenethyl­benzene­sulfonamide

**DOI:** 10.1107/S1600536809010927

**Published:** 2009-03-31

**Authors:** C. Suneel Manohar Babu, Helen P. Kavitha, R. Kavipriya, Jasmine P. Vennila, V. Manivannan

**Affiliations:** a Nicholas Piramal Research Centre, Nicholas Piramal India Limited, Mumbai 400 063, India; b Department of Chemistry, SRM University, Ramapuram, Chennai 600 089, India; c Department of Physics, Panimalar Institute of Technology, Chennai 600 095, India; d Department of Research and Development, PRIST University, Vallam, Thanjavur 613 403, India

## Abstract

In the title compound, C_14_H_13_Cl_2_NO_2_S, the dihedral angle between the phenyl ring and the benzene ring is 69.94 (9)°. Two short intra­molecular C—H⋯O contacts occur and a weak inter­molecular C—H⋯π inter­action is seen in the crystal.

## Related literature

For the biological activity of sulfonamides, see: Gadad *et al.* (2000 ▶); Misra *et al.* (1982 ▶); Zani & Vicini (1998 ▶); Maren (1976 ▶); Supuran *et al.* (1998 ▶); Renzi *et al.* (2000 ▶); Li *et al.* (1995 ▶); Yoshino *et al.* (1992 ▶). For related structures, see: Zhang *et al.* (2006 ▶); Andrighetti-Fröhner *et al.* (2007 ▶); For graph-set notation, see: Bernstein *et al.* (1995 ▶).
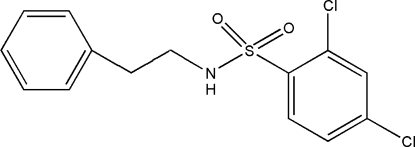


## Experimental

### 

#### Crystal data

C_14_H_13_Cl_2_NO_2_S
*M* *_r_* = 330.21
Orthorhombic, 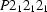

*a* = 5.5618 (5) Å
*b* = 10.9915 (8) Å
*c* = 25.045 (2) Å
*V* = 1531.0 (2) Å^3^
*Z* = 4
Mo *K*α radiation
μ = 0.56 mm^−1^
*T* = 295 K
0.20 × 0.18 × 0.12 mm

#### Data collection

Bruker Kappa APEXII CCD diffractometer
Absorption correction: multi-scan (**SADABS**; Sheldrick, 1996 ▶) *T*_min_ = 0.896, *T*_max_ = 0.936
10930 measured reflections
3511 independent reflections
2955 reflections with *I* > 2σ(*I*)
*R*_int_ = 0.026

#### Refinement

*R*[*F* ^2^ > 2σ(*F* ^2^)] = 0.040
*wR*(*F* ^2^) = 0.104
*S* = 1.05
3511 reflections
181 parameters
H-atom parameters constrained
Δρ_max_ = 0.27 e Å^−3^
Δρ_min_ = −0.24 e Å^−3^
Absolute structure: Flack (1983 ▶), 1455 Friedel pairs
Flack parameter: 0.04 (8)

### 

Data collection: *APEX2* (Bruker, 2004 ▶); cell refinement: *SAINT* (Bruker, 2004 ▶); data reduction: *SAINT*; program(s) used to solve structure: *SHELXS97* (Sheldrick, 2008 ▶); program(s) used to refine structure: *SHELXL97* (Sheldrick, 2008 ▶); molecular graphics: *PLATON* (Spek, 2009 ▶); software used to prepare material for publication: *SHELXL97*.

## Supplementary Material

Crystal structure: contains datablocks global, I. DOI: 10.1107/S1600536809010927/is2402sup1.cif

Structure factors: contains datablocks I. DOI: 10.1107/S1600536809010927/is2402Isup2.hkl

Additional supplementary materials:  crystallographic information; 3D view; checkCIF report

## Figures and Tables

**Table 1:** Hydrogen-bond geometry (Å, °)

*D*—H⋯*A*	*D*—H	H⋯*A*	*D*⋯*A*	*D*—H⋯*A*
C8—H8*B*⋯O2	0.97	2.51	2.953 (3)	108
C14—H14⋯O2	0.93	2.44	2.848 (3)	106
C6—H6⋯*Cg*1^i^	0.93	2.96	3.694 (3)	137

Symmetry code: (i) 
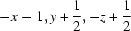
. *Cg*1 is the centroid of the C1–C6 ring.

**Fig. 1.**
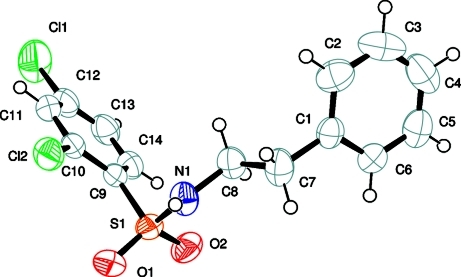
The molecular structure of (I), with atom labels and 50% probability displacement ellipsoids for non-H atoms.

## supplementary crystallographic information

### Comment

Sulfonamides have a variety of biological activities such as antibacterial (Gadad *et al.*, 2000; Misra *et al.*, 1982; Zani & Vicini, 1998), insulin releasing (Maren, 1976), carbonic anhydrase inhibitory (Supuran *et al.*, 1998; Renzi *et al.*, 2000), anti-inflammatory (Li *et al.*, 1995) and antitumor (Yoshino *et al.*, 1992) activities.

The geometric parameters in the title compound, (I), agree with the reported values of similar structure (Zhang *et al.*, 2006; Andrighetti-Fröhner *et al.*, 2007). The dihedral angle between the phenyl ring (C9—C14) and benzene ring (C1—C6) is 69.94 (9)°. The geometry around the S1 atom is distorted from a regular tetrahedron, with the largest deviations observed for O—S—O [O1—S1—O2 118.92 (14)°] and O—S—N [O1—S1—N1 107.87 (14) °] angles. The widening of the angles may be due to repulsive interactions between the two short S=O bonds.

The crystal structure is stabilized by weak intramolecular C—H···O interaction. The C8—H8B···O2 and C14—H14···O2 interactions each generate an S(5) graph set motif, and C8—H8B···O2 and C14—H14···O2 interactions together constitute a pair of bifurcated acceptor bonds, generating an *R*_2_^1^(8) motif (Bernstein *et al.*, 1995). The crystal packing is stabilized by a weak C—H···π (Table 1) interaction and a π–π interaction [*Cg*1···*Cg*2 (2 - *x*, 1/2 + *y*, 1/2 - *z*) distance of 4.3598 (18) Å; *Cg*1 and *Cg*2 are the centroids of rings C1—C6 and C9—C14, respectively].

### Experimental

About 1 g (8 mmol) of 2-phenylethyl amine is dissolved in 20 ml of dichloromethane. 1.3 g (16 mmol) of pyridine is added into the reaction mass. The above mixture is stirred for 5 min. To this, 2.41 g (9.8 mmol) of 2, 4-dichlorobenzene-1-sulfonyl chloride is added and heated to 35 - 40 ° C for 6 hrs. The reaction mass is then cooled to the room temperature and 20 ml of water is added to it. The aquous layer is separated. The organic layer is washed with 10% sodium chloride solution and dried over 2 g of anhydrous sodium sulfate. The excess solvent is removed under vacuum. The crude compound is purified through column chromatography using hexane and ethyl acetate as eluants.

### Refinement

H atoms were positioned geometrically and refined using riding model, with C—H = 0.93 Å and *U*_iso_(H) = 1.2*U*_eq_(C) for aromatic C—H, C—H = 0.97 Å and *U*_iso_(H) = 1.5*U*_eq_(C) for CH_2_, and N—H = 0.86 Å and *U*_iso_(H) = 1.2*U*_eq_(N).

### Crystal data

**Table:**

C_14_H_13_Cl_2_NO_2_S	*F*(000) = 680
*M**_r_* = 330.21	*D*_x_ = 1.433 Mg m^−^^3^
Orthorhombic, *P*2_1_2_1_2_1_	Mo *K*α radiation, λ = 0.71073 Å
Hall symbol: P 2ac 2ab	θ = 1.6–27.6°
*a* = 5.5618 (5) Å	µ = 0.56 mm^−^^1^
*b* = 10.9915 (8) Å	*T* = 295 K
*c* = 25.045 (2) Å	Block, white
*V* = 1531.0 (2) Å^3^	0.20 × 0.18 × 0.12 mm
*Z* = 4	

### Data collection

**Table:**

Bruker Kappa APEXII CCD diffractometer	3511 independent reflections
Radiation source: fine-focus sealed tube	2955 reflections with *I* > 2σ(*I*)
graphite	*R*_int_ = 0.026
ω and φ scans	θ_max_ = 27.6°, θ_min_ = 2.5°
Absorption correction: multi-scan (*SADABS*; Sheldrick, 1996)	*h* = −7→7
*T*_min_ = 0.896, *T*_max_ = 0.936	*k* = −8→13
10930 measured reflections	*l* = −32→32

### Refinement

**Table:**

Refinement on *F*^2^	Secondary atom site location: difference Fourier map
Least-squares matrix: full	Hydrogen site location: inferred from neighbouring sites
*R*[*F*^2^ > 2σ(*F*^2^)] = 0.040	H-atom parameters constrained
*wR*(*F*^2^) = 0.104	*w* = 1/[σ^2^(*F*_o_^2^) + (0.0546*P*)^2^ + 0.264*P*] where *P* = (*F*_o_^2^ + 2*F*_c_^2^)/3
*S* = 1.05	(Δ/σ)_max_ < 0.001
3511 reflections	Δρ_max_ = 0.27 e Å^−^^3^
181 parameters	Δρ_min_ = −0.23 e Å^−^^3^
0 restraints	Absolute structure: Flack (1983), 1455 Friedel pairs
Primary atom site location: structure-invariant direct methods	Flack parameter: 0.04 (8)

### Special details

**Table:**

Geometry. All e.s.d.'s (except the e.s.d. in the dihedral angle between two l.s. planes) are estimated using the full covariance matrix. The cell e.s.d.'s are taken into account individually in the estimation of e.s.d.'s in distances, angles and torsion angles; correlations between e.s.d.'s in cell parameters are only used when they are defined by crystal symmetry. An approximate (isotropic) treatment of cell e.s.d.'s is used for estimating e.s.d.'s involving l.s. planes.
Refinement. Refinement of *F*^2^ against ALL reflections. The weighted *R*-factor *wR* and goodness of fit *S* are based on *F*^2^, conventional *R*-factors *R* are based on *F*, with *F* set to zero for negative *F*^2^. The threshold expression of *F*^2^ > σ(*F*^2^) is used only for calculating *R*-factors(gt) *etc*. and is not relevant to the choice of reflections for refinement. *R*-factors based on *F*^2^ are statistically about twice as large as those based on *F*, and *R*- factors based on ALL data will be even larger.

### Fractional atomic coordinates and isotropic or equivalent isotropic displacement parameters (Å^2^)

**Table:**

	*x*	*y*	*z*	*U*_iso_*/*U*_eq_	
S1	0.77658 (10)	0.02339 (6)	0.22502 (2)	0.04851 (16)	
Cl2	1.21110 (11)	−0.02059 (7)	0.30970 (3)	0.06133 (19)	
Cl1	0.8648 (3)	0.32987 (9)	0.43460 (4)	0.1235 (5)	
C1	1.1601 (5)	0.2812 (2)	0.07918 (9)	0.0492 (6)	
C2	1.3486 (6)	0.3577 (3)	0.08766 (14)	0.0721 (9)	
H2	1.4664	0.3368	0.1123	0.087*	
C3	1.3671 (7)	0.4658 (4)	0.06019 (18)	0.0914 (11)	
H3	1.4972	0.5170	0.0664	0.110*	
C4	1.1958 (8)	0.4980 (3)	0.02405 (14)	0.0816 (10)	
H4	1.2091	0.5707	0.0053	0.098*	
C5	1.0062 (7)	0.4235 (3)	0.01560 (11)	0.0727 (9)	
H5	0.8878	0.4457	−0.0087	0.087*	
C6	0.9872 (5)	0.3153 (3)	0.04272 (10)	0.0578 (7)	
H6	0.8564	0.2646	0.0364	0.069*	
C7	1.1389 (6)	0.1609 (3)	0.10805 (10)	0.0649 (8)	
H7A	1.0433	0.1056	0.0866	0.078*	
H7B	1.2979	0.1257	0.1119	0.078*	
C8	1.0261 (5)	0.1724 (2)	0.16216 (9)	0.0541 (6)	
H8A	1.1250	0.2242	0.1846	0.065*	
H8B	0.8690	0.2100	0.1588	0.065*	
C9	0.8116 (4)	0.1116 (2)	0.28395 (9)	0.0413 (5)	
C10	0.9959 (5)	0.0905 (2)	0.32017 (9)	0.0464 (5)	
C11	1.0144 (6)	0.1593 (3)	0.36615 (11)	0.0625 (7)	
H11	1.1390	0.1460	0.3902	0.075*	
C12	0.8444 (8)	0.2484 (2)	0.37570 (12)	0.0683 (9)	
C13	0.6653 (7)	0.2725 (3)	0.34033 (12)	0.0649 (8)	
H13	0.5550	0.3341	0.3472	0.078*	
C14	0.6490 (5)	0.2044 (2)	0.29421 (11)	0.0527 (6)	
H14	0.5277	0.2209	0.2697	0.063*	
O1	0.7876 (4)	−0.10180 (17)	0.23927 (8)	0.0660 (5)	
O2	0.5657 (3)	0.0685 (2)	0.19918 (7)	0.0652 (5)	
N1	1.0011 (4)	0.05205 (19)	0.18713 (7)	0.0525 (5)	
H1	1.1075	−0.0032	0.1813	0.063*	

### Atomic displacement parameters (Å^2^)

**Table:**

	*U*^11^	*U*^22^	*U*^33^	*U*^12^	*U*^13^	*U*^23^
S1	0.0474 (3)	0.0475 (3)	0.0506 (3)	−0.0037 (3)	−0.0090 (2)	0.0003 (3)
Cl2	0.0503 (3)	0.0567 (4)	0.0770 (4)	0.0050 (3)	−0.0114 (3)	0.0147 (3)
Cl1	0.2342 (16)	0.0666 (5)	0.0696 (5)	0.0090 (8)	−0.0224 (7)	−0.0190 (4)
C1	0.0556 (14)	0.0533 (14)	0.0387 (11)	0.0056 (12)	0.0057 (10)	−0.0047 (10)
C2	0.0584 (17)	0.077 (2)	0.081 (2)	0.0036 (16)	−0.0144 (15)	−0.0071 (16)
C3	0.071 (2)	0.077 (2)	0.127 (3)	−0.027 (2)	0.006 (2)	−0.016 (2)
C4	0.112 (3)	0.0536 (18)	0.079 (2)	−0.0024 (19)	0.033 (2)	0.0067 (15)
C5	0.088 (2)	0.075 (2)	0.0555 (16)	0.015 (2)	−0.0057 (16)	0.0062 (14)
C6	0.0588 (16)	0.0628 (17)	0.0519 (13)	−0.0051 (14)	−0.0074 (12)	0.0016 (12)
C7	0.089 (2)	0.0573 (17)	0.0487 (14)	0.0127 (16)	0.0044 (14)	0.0025 (12)
C8	0.0655 (16)	0.0460 (14)	0.0509 (13)	0.0075 (13)	0.0051 (12)	−0.0006 (11)
C9	0.0406 (11)	0.0402 (12)	0.0431 (11)	−0.0074 (9)	−0.0018 (9)	0.0066 (9)
C10	0.0468 (13)	0.0409 (13)	0.0514 (12)	−0.0059 (10)	−0.0047 (11)	0.0113 (10)
C11	0.083 (2)	0.0507 (16)	0.0538 (14)	−0.0166 (15)	−0.0199 (14)	0.0106 (12)
C12	0.111 (3)	0.0363 (14)	0.0576 (15)	−0.0069 (15)	−0.0049 (18)	0.0012 (11)
C13	0.084 (2)	0.0424 (15)	0.0684 (17)	0.0063 (14)	0.0066 (16)	0.0037 (13)
C14	0.0527 (14)	0.0469 (14)	0.0584 (14)	0.0022 (11)	0.0003 (11)	0.0085 (11)
O1	0.0804 (14)	0.0448 (10)	0.0729 (12)	−0.0147 (10)	−0.0121 (11)	0.0008 (8)
O2	0.0520 (10)	0.0814 (14)	0.0622 (11)	−0.0011 (9)	−0.0156 (9)	−0.0014 (10)
N1	0.0615 (13)	0.0455 (12)	0.0505 (11)	0.0137 (10)	0.0049 (10)	0.0022 (9)

### Geometric parameters (Å, °)

**Table:**

S1—O1	1.423 (2)	C6—H6	0.9300
S1—O2	1.429 (2)	C7—C8	1.499 (4)
S1—N1	1.600 (2)	C7—H7A	0.9700
S1—C9	1.777 (2)	C7—H7B	0.9700
Cl2—C10	1.730 (3)	C8—N1	1.470 (3)
Cl1—C12	1.730 (3)	C8—H8A	0.9700
C1—C2	1.361 (4)	C8—H8B	0.9700
C1—C6	1.378 (4)	C9—C14	1.387 (3)
C1—C7	1.512 (4)	C9—C10	1.388 (3)
C2—C3	1.376 (5)	C10—C11	1.382 (4)
C2—H2	0.9300	C11—C12	1.382 (5)
C3—C4	1.361 (5)	C11—H11	0.9300
C3—H3	0.9300	C12—C13	1.359 (5)
C4—C5	1.351 (5)	C13—C14	1.379 (4)
C4—H4	0.9300	C13—H13	0.9300
C5—C6	1.374 (4)	C14—H14	0.9300
C5—H5	0.9300	N1—H1	0.8600
			
O1—S1—O2	119.00 (13)	H7A—C7—H7B	107.8
O1—S1—N1	107.80 (12)	N1—C8—C7	110.4 (2)
O2—S1—N1	107.69 (11)	N1—C8—H8A	109.6
O1—S1—C9	108.35 (11)	C7—C8—H8A	109.6
O2—S1—C9	106.06 (11)	N1—C8—H8B	109.6
N1—S1—C9	107.44 (11)	C7—C8—H8B	109.6
C2—C1—C6	118.2 (3)	H8A—C8—H8B	108.1
C2—C1—C7	121.8 (3)	C14—C9—C10	118.9 (2)
C6—C1—C7	120.0 (3)	C14—C9—S1	118.93 (18)
C1—C2—C3	120.9 (3)	C10—C9—S1	122.15 (19)
C1—C2—H2	119.6	C11—C10—C9	120.5 (3)
C3—C2—H2	119.6	C11—C10—Cl2	117.5 (2)
C4—C3—C2	120.3 (3)	C9—C10—Cl2	122.02 (19)
C4—C3—H3	119.8	C12—C11—C10	118.8 (3)
C2—C3—H3	119.8	C12—C11—H11	120.6
C5—C4—C3	119.5 (3)	C10—C11—H11	120.6
C5—C4—H4	120.2	C13—C12—C11	121.8 (3)
C3—C4—H4	120.2	C13—C12—Cl1	120.2 (3)
C4—C5—C6	120.5 (3)	C11—C12—Cl1	118.0 (3)
C4—C5—H5	119.8	C12—C13—C14	119.2 (3)
C6—C5—H5	119.8	C12—C13—H13	120.4
C5—C6—C1	120.6 (3)	C14—C13—H13	120.4
C5—C6—H6	119.7	C13—C14—C9	120.7 (3)
C1—C6—H6	119.7	C13—C14—H14	119.6
C8—C7—C1	113.0 (2)	C9—C14—H14	119.6
C8—C7—H7A	109.0	C8—N1—S1	120.22 (17)
C1—C7—H7A	109.0	C8—N1—H1	119.9
C8—C7—H7B	109.0	S1—N1—H1	119.9
C1—C7—H7B	109.0		
			
C6—C1—C2—C3	−0.6 (5)	C14—C9—C10—C11	1.1 (3)
C7—C1—C2—C3	178.7 (3)	S1—C9—C10—C11	−178.63 (19)
C1—C2—C3—C4	0.2 (6)	C14—C9—C10—Cl2	−178.26 (18)
C2—C3—C4—C5	0.5 (6)	S1—C9—C10—Cl2	2.0 (3)
C3—C4—C5—C6	−0.8 (5)	C9—C10—C11—C12	0.9 (4)
C4—C5—C6—C1	0.4 (5)	Cl2—C10—C11—C12	−179.7 (2)
C2—C1—C6—C5	0.3 (4)	C10—C11—C12—C13	−2.3 (5)
C7—C1—C6—C5	−179.0 (3)	C10—C11—C12—Cl1	177.8 (2)
C2—C1—C7—C8	84.4 (4)	C11—C12—C13—C14	1.6 (5)
C6—C1—C7—C8	−96.4 (3)	Cl1—C12—C13—C14	−178.5 (2)
C1—C7—C8—N1	177.6 (2)	C12—C13—C14—C9	0.5 (4)
O1—S1—C9—C14	−130.6 (2)	C10—C9—C14—C13	−1.8 (4)
O2—S1—C9—C14	−1.7 (2)	S1—C9—C14—C13	177.9 (2)
N1—S1—C9—C14	113.21 (19)	C7—C8—N1—S1	−146.0 (2)
O1—S1—C9—C10	49.2 (2)	O1—S1—N1—C8	174.72 (19)
O2—S1—C9—C10	178.00 (18)	O2—S1—N1—C8	45.2 (2)
N1—S1—C9—C10	−67.0 (2)	C9—S1—N1—C8	−68.7 (2)

### Hydrogen-bond geometry (Å, °)

**Table:**

*D*—H···*A*	*D*—H	H···*A*	*D*···*A*	*D*—H···*A*
C8—H8B···O2	0.97	2.51	2.953 (3)	108
C14—H14···O2	0.93	2.44	2.848 (3)	106
C6—H6···Cg1^i^	0.93	2.96	3.694 (3)	137

Symmetry codes: (i) −*x*−1, *y*+1/2, −*z*+1/2.
